# Cassava mosaic disease and its whitefly vector in Cameroon: Incidence, severity and whitefly numbers from field surveys

**DOI:** 10.1016/j.cropro.2022.106017

**Published:** 2022-08

**Authors:** Oumar Doungous, Boutou Masky, Dopgima L. Levai, Joseph A.L. Bahoya, Emile Minyaka, Jacques F. Mavoungou, J. Musembi Mutuku, Justin S. Pita

**Affiliations:** a The Central and **W**est **A**frican **V**irus **E**pidemiology (WAVE), Biotechnology Laboratory, Ekona Regional Research Centre, Institute of Agricultural Research for Development, PMB 25, Buea, Cameroon; b Institut Universitaire de Technologie/Faculté des Sciences, Université de Douala, BP 24157, Douala, Cameroon; c Institut de Recherches Agronomiques et Forestières (IRAF), The Central and **W**est **A**frican **V**irus **E**pidemiology (WAVE), Libreville, Gabon; d The Central and **W**est **A**frican **V**irus **E**pidemiology (WAVE), Pôle Scientifique et d'Innovation de Bingerville, Université Félix Houphouët-Boigny, BP V34, Abidjan 01, Republic of Côte d'Ivoire

**Keywords:** Manihot esculenta, *Bemisia tabaci*, Cassava mosaic geminiviruses, Cassava database

## Abstract

Cassava plays a key role in the food security and economy of Cameroon, but its production is constrained by cassava mosaic disease (CMD). However, comprehensive surveys of CMD in Cameroon have been lacking. This study aimed at evaluating the current status of CMD and its whitefly vector. Field surveys were conducted in 2020 using a sampling, diagnostics and data storage protocol that has been harmonized across 10 West and Central African countries for ease of comparison. Thirty plants per field were assessed for CMD severity, whitefly abundance and source of infection. Surveys were conducted in 343 fields and confirmed the presence of CMD in all 10 regions of Cameroon. Among the 10,057 assessed plants, 33.07% were deemed healthy (asymptomatic). At the field level, only 6.7% fields were found to be healthy. The mean CMD incidence across the country was 66.93%, and the mean severity score was 2.28. The main mode of infection was likely through contaminated cuttings. The mean whitefly count per plant was 5.78. This study is the first countrywide survey of CMD in Cameroon and provides insights that can be useful for improving the country's CMD intervention and management strategies.

## Highlights

• First countrywide survey of cassava mosaic disease (CMD) in Cameroon (343 fields).
• CMD and whiteflies were recorded in all 10 regions of the country.
• Mean CMD incidence was 66.93%, and mean whitefly count was 5.78 per plant.
• Diseased cuttings were the main source of CMD infection.

## 1 Introduction

Cassava viruses threaten food security and income for millions of Africans who depend on cassava (*Manihot esculenta*) and cassava products for their food and livelihood. Cassava mosaic disease (CMD) and cassava brown streak disease are the two most important constraints affecting cassava production in Africa (Legg et al., 2014a; Patil et al., 2015). In Cameroon, cassava is cultivated in all 10 regions of the country, the largest producers being East, Centre and South Regions (Njukwe et al., 2013; INS, 2017), but the presence of CMD is a constraint in all cassava-growing areas (Akinbade et al., 2010). The disease is caused by cassava mosaic geminiviruses (CMGs), which are geminiviruses of the genus *Begomovirus*, family *Geminiviridae*. Out of the nine CMG species found in Africa (Soro et al., 2021), previous studies confirmed the occurrence of African cassava mosaic virus (ACMV), East African cassava mosaic Cameroon virus (EACMCV) and East African cassava mosaic virus (EACMV) in CMD etiology in Cameroon (Fondong et al., 2000; Akinbade et al., 2010). The Ugandan variant of EACMV (EACMV-UG), a very virulent recombinant strain responsible for the severe CMD epidemics in East and Central Africa, was also reported in Cameroon, in the East Region (Akinbade et al., 2010). These CMG species and strains frequently occur in mixed infections and their synergy results in more severe crop symptoms (Fondong et al., 2000; Chikoti et al., 2019).

The CMGs are transmitted by members of the cryptic whitefly species complex *Bemisia tabaci* (Gennadius) (Aleyrodidae: Hemiptera). The viruses are spread through infected cuttings originating from diseased plants and used as planting material or by infected whiteflies feeding on the plants (Zinga et al., 2013; Legg et al., 2014b; MacFadyen et al., 2018). In addition to its ability to transmit CMGs and cassava brown streak viruses (Maruthi et al., 2017), *B. tabaci* also damages cassava through direct feeding which causes chlorotic mottling and twisting or curling, particularly on upper leaves (Bellotti and Arias, 2001; Legg et al., 2014b). They produce honeydew that falls onto the lower leaves, leading to black sooty mold colonization and the subsequent reduction of photosynthesis (Bellotti and Arias, 2001; Legg et al., 2014b).

Symptoms of CMD are generally visible on the leaves, and their extent may vary according to the virus species or strain, the environment and the cassava plant host (Legg and Thresh, 2003). Infected plants express a range of symptoms and the most typical consist of a yellow or pale green chlorotic mottling on leaves, commonly accompanied by distortion and crumpling (Fauquet and Fargette, 1990; Legg and Thresh, 2003). In the case of mild symptoms, leaf chlorosis, leaf distortion or malformation may be absent on some leaves. Severe symptoms are associated with plant stunting, or necrosis and shriveling of petioles (Fauquet and Fargette, 1990; Legg and Thresh, 2003).

Cameroon's economy remains highly dependent on its agricultural sector, which employs about 60% of the national active labor force (Abia et al., 2016; INS 2017). Agriculture contributes around 15.33% to the gross domestic product and 24.49% of merchandise exports (Mouafor et al., 2016). The current government strategy for agricultural development revolves around a more intensive-based agricultural sector, which is stimulated by dynamic and growth-generating value chains that provide employment – this includes cassava. Even though cassava is produced mostly by smallholders in Cameroon, the country produced about 4,858,329 tonnes of cassava in 2020, placing the country 13th in the world for its contribution of about 1.6% of world production (FAOSTAT, 2020). In 2020, the extent of cassava cultivation was almost 329,371 ha (FAOSTAT, 2020) with an average yield of 14.75 t/ha. Cassava is also used as a source of income generation. It provides higher income to growers well over that for rice and maize, its two major competitors in Cameroon (Mvodo and Liang, 2012).

Cassava yields in Cameroon are low, based on the cultivated areas and the existence of suitable climate conditions. Productivity could be increased if improved varieties, disease-free planting materials, and good management practices were used. As indicated by Akinbade et al. (2010) and Tize et al. (2021), CMD is a serious constraint to cassava production, leading to heavy yield losses. Cassava is grown in a wide range of regions in Cameroon – from the equatorial rainforest in the south to the subtropical semi-arid in the north – thus, cassava variety, variation of cultural practices and environmental factors at different sites may have a bearing on CMD incidence and severity and the vector abundance. Good quality survey data for CMD are still lacking in Cameroon. To our knowledge, the last published surveys conducted by the International Institute of Tropical Agriculture focused only on the virus diagnosis in the Centre, South and East Regions (Akinbade et al., 2010). However, countrywide surveys coupled with the use of an efficient data collection method are needed to provide recommendations for the control initiatives and effective management of cassava virus diseases.

Therefore, this study aimed to analyze the current status of CMD and its whitefly vector in Cameroon. It was carried out under the project Central and West African Virus Epidemiology (WAVE) for food security. The WAVE program addresses virus diseases that infect cassava, yams and sweet potato by empowering smallholder farmers and appropriate stakeholders with appropriate technologies to better manage these diseases in 10 countries across West and Central Africa, including Cameroon. We conducted field surveys in all cassava-growing areas in Cameroon, determined the CMD incidence, severity and whitefly numbers, as well as possible relationships between associated-CMD variables (incidence, severity and vector abundance), and field altitude using a comprehensive, multi-dimensional cassava database.

## 2 Materials and methods

### 2.1 Location

Administratively Cameroon is divided into 10 regions and 58 divisions which can also be distinguished by dominant climatic and vegetative features (Table 1).

**Table 1:** Description of regions of Cameroon.

Region	Average rainfall (mm)	Average mean temperature (°C)	Annual relative humidity (%)	Predominant agro-ecological zone
Far-North	717.49	28.18	52	I
North	1155.72	26.51	52	I
Adamawa	1508.35	23.36	59	II
East	1552.58	24.31	84	V
West	1949.45	22.70	87	III
North-West	1897.01	23.01	87	III
Littoral	2371.29	25.93	87	IV
South-West	2362.54	25.12	87	IV
South	1861.32	24.59	84	V
Centre	1700.45	24.62	84	V

Observed average annual rainfall and mean temperature were for the period 1991–2020 (Harris et al., 2020).

Source (average humidity): https://www.timeanddate.com, accessed March 29, 2022.

I = Sudano-Sahelian, II = High Guinea Savannah, III = Western Highlands, IV = Humid Forest (monomodal rainfall), V = Humid Forest (bimodal rainfall).

### 2.2 Survey

**Fig. 1:**
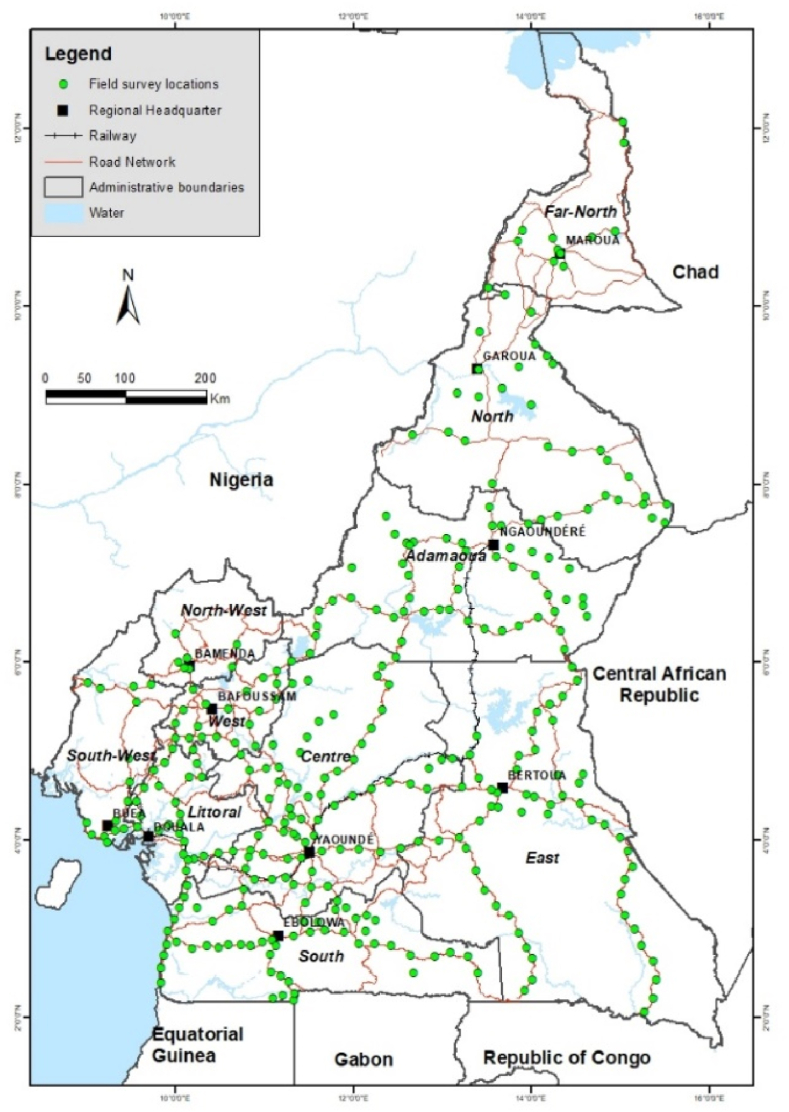
Regions and field locations surveyed in Cameroon.

Using WAVE's harmonized sampling methods and standardized diagnostic protocols (Sseruwagi et al., 2004; Eni et al., 2021; Soro et al., 2021), field surveys were conducted in all 10 regions of Cameroon (Fig. 1). This involved collecting data and samples (cassava leaves and whitefly vector) from cassava plants at 3–6 months after planting. A total of 343 fields were randomly chosen and surveyed from the 10 regions.

The survey route was along marked roads to villages, and fields within the villages were sampled. Distances between survey sites varied depending on the availability of cassava farms in each area but the minimum distance was generally 20–30 km. Before entering farms, verbal permission to enter and work in their fields was requested by the survey team from the field owners or their representatives.

### 2.3 Data recording and storage

At each survey site, data were recorded using a tablet with the survey software iForm Zerion (version 9.1.6) developed by the University of Cambridge, UK's Epidemiological Modelling Group. Data recorded at each site comprised the name and administrative unit of the locality, geographical coordinates (latitude and longitude), altitude of sampling sites, the CMD symptoms observed, and whitefly counts. Additional information on cassava variety, date and time, field size, planting type and distance between surveyed fields was also recorded. The recorded data were uploaded to iForm's cloud-based database and then integrated into the WAVE Cube – the latter is a novel, multi-dimensional database for the storage of cassava data that was developed specifically for cassava data storage within the WAVE program.

A total of 30 cassava plants were assessed along 2 diagonals in an X shape (15 plants chosen randomly on each diagonal). The distance between plants assessed varied depending on the size of the field (0.3–2 ha). On each selected plant, observations were made on CMD severity, whitefly abundance and where the plant was infected – the source of infection was determined as either from cuttings or by the vector.

The severity of the symptoms was recorded using the standard scale of 1–5: 1 = No symptoms; 2 = Mild chlorotic pattern on entire leaflets with no leaf distortion or size reduction of leaflets; 3 = Strong mosaic pattern on the entire leaf, with some distortion of lower one-third of leaflets, no size reduction; 4 = Severe mosaic distortion of two-thirds of leaflets and general reduction in leaf size; and 5 = Very severe symptoms, including severe chlorosis, leaves distortion and plant stunting (Hahn et al., 1980).

The CMD incidence was calculated as the percentage of CMD-symptomatic plants out of the total plants assessed. The incidence was then visually categorized into five percentage bands: fields with 0 incidence were recorded as *Healthy*; >0–25% as *Low* incidence; >25–50% as *Medium* incidence; >50–75% as *High* incidence; and >75–100% as *Very High* incidence.

The whitefly population was estimated by counting the number of whiteflies on the top five fully expanded leaves of each plant. The mean of whiteflies per plant was calculated as the total number of whiteflies recorded on 30 plants divided by 30. About 100 adult whiteflies per surveyed field were collected randomly from cassava plants and stored in screw cap Eppendorf tubes containing 90% ethanol. Tubes were labeled and sealed with parafilm. The whitefly samples will be used later for vector biotyping, which is beyond the scope of this study.

The source of infection was determined according to Sseruwagi et al. (2004): whitefly-derived infections cause disease symptoms only on the upper leaves, whereas cutting-derived infections also cause symptoms on the lowest first formed leaves.

At each field, samples of cassava leaves displaying different severity scores (mild symptoms, severe symptoms and, if possible asymptomatic from healthy plants) were collected and conserved in a plant press. Collected leaf samples were labeled with barcodes for later use in virus characterization.

### 2.4 Data visualization and analysis

Data from the WAVE Cube can be accessed for examination at various levels: field, division, region and country. The data can be selected and visualized in different formats, e.g. table, graph or histogram. Maps can be generated using Microsoft's PowerBI modeling tool using the coordinates recorded in the Cube.

Statistical analyses were performed using SPSS software (version 25 for Windows, SPSS Inc., Chicago, IL, USA). Pairwise correlations between variables were examined using Spearman's rank correlation analyses. The map of Cameroon showing the regions and geographical distribution of the surveyed fields was developed using ArcGIS version 10.8.1.

## 3 Results

### 3.1 CMD status in surveyed fields

At the plant level, 3326 out of 10,057 assessed plants were healthy (asymptomatic) and received a score of 1. The highest severity score (5) was recorded on only 23 plants. There were 5111 plants assigned a severity score of 2, which was the most frequently observed severity score (Table 2).

**Table 2:** Number of plants for CMD severity scores and infection sources.

Severity scores	Number of plants	Cutting-derived infection	Whitefly-derived infection
Severity 1	3326	–	–
Severity 2	5111	4817	294
Severity 3	1373	1272	101
Severity 4	224	197	27
Severity 5	23	15	8
Total	10,057	6301	430

**Fig. 2:**
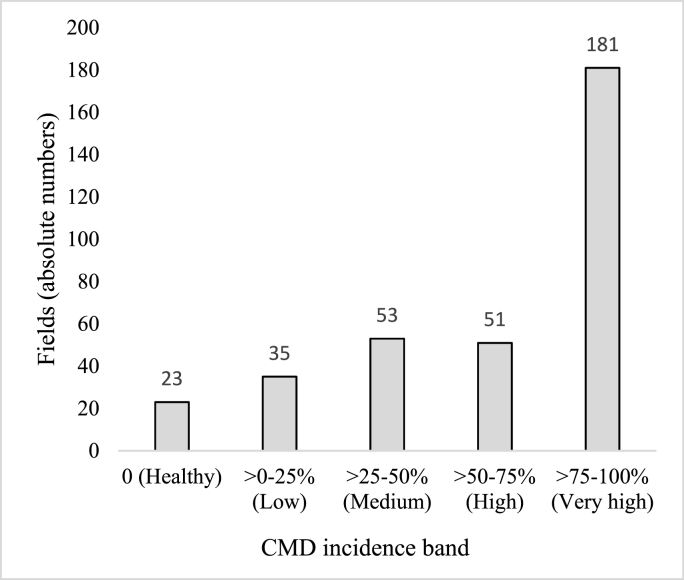
Number of fields within CMD incidence bands.

Out of 343 fields, 23 were characterized as healthy, and received a score of 0. Although these fields were scattered across the country, nine were in the North Region and eight in the Adamawa Region. Two healthy fields were in the East Region, and a single field was located in each of the Centre, Littoral, West and South Regions. However, the mean CMD incidence across the country was 66.93%, which is considered high. This likely resulted from many of the fields having high incidence scores, with 181 fields showing incidence scores exceeding 75% (Fig. 2). A weak, positive correlation was found between CMD incidence and severity score (*P* ˂ 0.01) (Table 3).

**Table 3:** Pairwise Spearman rank correlation coefficients r_s_ (normal text) and *P*-values (italics).

Parameters	CMD incidence	CMD severity	Whitefly abundance	Altitude
CMD incidence	1 …	0.303** 0.000	0.278** 0.000	−0.104 *0.055*
CMD severity	… …	1 …	0.014 *0.792*	−0.024 *0.656*
Whitefly abundance	… …	… …	1 …	−0.263** 0.000
Altitude	…	…	…	1

**, significant at *P* < 0.01 (two-tailed), n = 343.

The mean CMD incidence ranged from 30% for the North Region to 89.14% for the South Region (Table 4). The low CMD incidence of the North was likely because this region had the highest number of healthy fields, and all four divisions surveyed in this region had an incidence below 37.67%. Nonetheless, among all divisions, the lowest CMD incidence of 6.60% was recorded for Faro et Deo Division in the Adamawa Region. The very high CMD incidence for the South Region was consistent among divisions in this region. In contrast, among all divisions, the highest mean CMD incidence (100%) was recorded for Nyong et Mfoumou (Centre), Bamboutos (West) and Menchum (North-West).

**Table 4:** Number of surveyed fields per region, CMD severity, CMD incidence and whitefly number (by division, within region).

Region	Number of fields	Division	CMD severity mean	CMD incidence mean	Whitefly number
Adamawa	50		2.31	54.07	4.41
		Djerem	2.27	57.04	5.57
		Faro et Deo	2.08	6.60	0.29
		Mayo-Banyo	2.13	61.67	0.49
		Mberé	2.46	72.42	13.55
		Vina	2.32	55.56	0.89
Centre	67		2.35	81.69	3.60
		Haute-Sanaga	2.72	74.81	1.87
		Lekié	2.43	89.91	1.39
		Mbam et Inoubou	2.35	92.22	2.35
		Mbam et Kim	2.10	61.85	6.74
		Mefou et Afamba	2.41	98.33	2.00
		Mefou et Akono	2.21	96.67	1.65
		Nyong et Kéllé	2.21	90.00	4.56
		Nyong et Mfoumou	2.37	100.00	2.12
		Nyong et So	2.46	95.24	3.30
East	56		2.44	60.55	4.02
		Boumba et Ngoko	2.42	73.33	3.01
		Haut-Nyong	2.43	60.21	3.00
		Kadei	2.36	69.26	4.07
		Lom et Djerem	2.52	49.12	5.45
Far-North	14		2.81	39.85	1.97
		Diamaré	2.92	50.23	1.74
		Logone et Chari	2.17	10.00	1.55
		Mayo-Danay	2.50	13.33	2.90
		Mayo-Sava	2.83	40.00	1.20
		Mayo-Tsanaga	2.56	45.00	3.13
Littoral	26		2.23	80.61	17.77
		Moungo	2.15	52.11	6.79
		Nkam	2.19	86.19	48.63
		Sanaga-Maritime	2.30	96.33	5.05
		Wouri	2.04	86.67	6.90
North	33		2.28	30.00	2.18
		Bénoué	2.29	37.67	5.33
		Faro	2.36	12.22	2.06
		Mayo-Louti	2.32	36.67	2.98
		Mayo-Rey	2.25	27.96	0.37
North-West	7		2.28	89.01	20.60
		Bui	2.00	70.00	3.03
		Menchum	2.03	100.00	6.33
		Momo	2.40	91.67	28.89
		Ngo-Ketunjia	3.00	50.00	1.00
South	54		2.13	89.14	4.78
		Dja et Lobo	2.10	84.96	3.88
		Mvila	2.08	97.96	3.82
		Ocean	2.18	89.41	6.44
		Vallée du Ntem	2.12	90.37	3.70
South-West	16		2.10	73.68	19.45
		Fako	2.05	48.22	9.02
		Manyu	2.10	95.33	18.81
		Meme	2.17	93.33	43.37
West	20		2.06	59.83	2.73
		Bamboutos	2.00	100.00	2.73
		Haut Nkam	2.17	46.00	3.73
		Menoua	2.00	35.00	4.48
		Mifi	2.00	23.33	2.33
		Ndé	2.11	76.67	4.23
		Noun	2.03	68.89	1.49
Mean	343		2.28	66.93	5.78

At region or division levels, CMD severity mean: sum of all plant severity values greater than 1 out of the count of infected plants.

CMD incidence mean: percentage of the total count of infected plants out of total count of plants.

Whitefly number: mean number of whitefly per plant.

The mean CMD severity across the country was 2.28. The West Region had the lowest mean CMD severity (2.06). This is unsurprising because three divisions out of the four (Bamboutos, Menoua and Mifi) with the lowest CMD severity mean (2) were located in the West Region. Bui in the North-West Region was the only division not in the West Region that displayed the lowest CMD severity mean. Although the Far-North was the region with the highest mean CMD severity (2.81), Ngo-Ketunjia, the division with highest severity mean (3.0) was located in the North-West Region.

### 3.2 Whitefly population

Mean whitefly counts varied with survey site (Table 4). The mean whitefly count per plant across the country was 5.78. At the regional level, the mean whitefly count varied from 1.97 in the Far-North Region to 20.6 in the North-West Region. However, greater variation was observed among divisions. The highest whitefly mean count of 48.63 was recorded for Nkam Division (Littoral Region) while Faro et Deo in the Adamawa Region had the lowest mean (0.29). Field-level data showed four fields with whitefly counts higher than 50 located in the Littoral, South-West and North-West Regions. The field with the highest whitefly mean count per plant of 297 was located in the Littoral Region (Supplementary material 1).

### 3.3 Relationship between whitefly populations and other variables

**Fig. 3:**
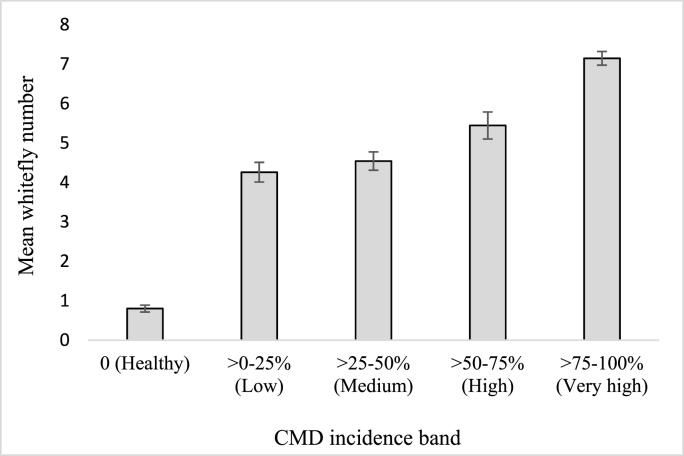
Mean number of whiteflies per plant by CMD incidence band. Bars represent standard error of mean (SEM).

Whitefly counts had weak, positive correlations (*P* ˂ 0.01) with CMD incidence (Table 3). The lowest whitefly count was observed in healthy fields, and CMD incidence increased with increasing whitefly counts (Fig. 3). The highest whitefly mean count of 7.14 was recorded in fields within the very high CMD incidence band.

**Fig. 4:**
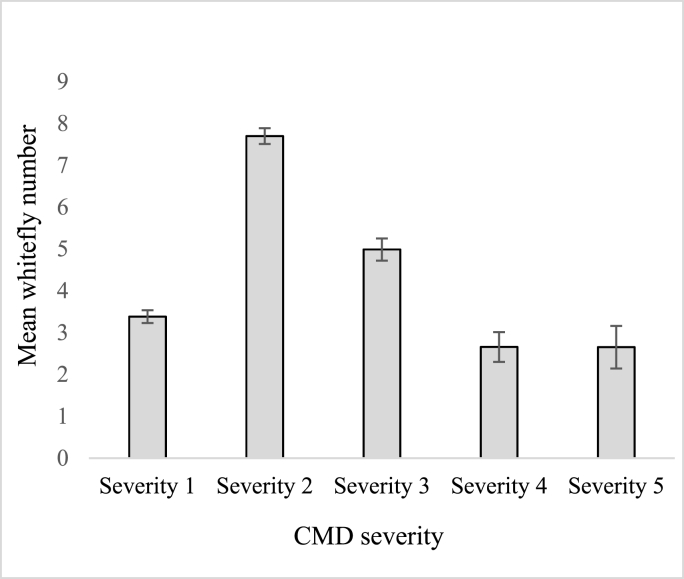
Mean number of whiteflies per plant within each CMD severity category. Bars represent SEM.

Although there were no correlations between CMD severity and whitefly abundance (*P* = 0.792) (Table 3), the highest whitefly mean count (7.69) was recorded on plants with CMD severity score of 2 (Fig. 4). As CMD severity increased, the whitefly population gradually decreased. The lowest whitefly mean count (2.65) was recorded on plants with the highest severity score.

Surveyed fields were located at different altitudes varying from the coastal regions at 0 m above sea level (ASL) up to the Western Highlands at 1703 m ASL (Supplementary material 1). Statistical analysis revealed a significant weak negative correlation between altitude and whitefly abundance (*P* ˂ 0.01) (Table 3).

### 3.4 Source of CMD infection

The visual assessment showed that the main mode of CMD infection was through contaminated cuttings (Table 2). This accounted for approximately 93.6% of diseased plants across all surveyed areas. Infection of the remaining diseased plants (6.4%) may have been caused by the insect vector *B. tabaci*.

## 4 Discussion

This study is the first countrywide survey of CMD in Cameroon. The high mean CMD incidence in the country may be associated with many factors. Most farmers still use low-yield local landraces that are highly susceptible to CMD, and often do not follow good management practices. This is unsurprising since most farmers do not consider CMD a serious constraint (Poubom et al., 2005), or do not know the cause or the vector of the disease, as also reported in other African countries (Chikoti et al., 2016; Houngue et al., 2018).

A few healthy farms were found, mostly in the North and Adamawa Regions. The Adamawa, North and Far-North Regions generally had low CMD incidence and severity compared to the other regions. The low level of CMD found may be because farmers in these regions have benefited from and adopted improved varieties. Fotso et al. (2018) showed in field experiments that these varieties had less than 16% CMD infection across different environments of Cameroon. The intensification of cassava cultivation in these regions is recent compared to the other regions (Kegah et al., 2018). As these farmers cultivate cassava in large areas, this required substantial investment that often leads to adoption of better management practices against cassava pests and diseases.

In this survey, the adult whitefly numbers per plant averaged 5.78. This figure is higher than those recently reported in Ghana (Oppong et al., 2021) and Burkina Faso (Soro et al., 2021). The high whitefly population could be attributed to the differences in factors such as cassava cultivars, whitefly genotypes, cultural practices, or climate change that have been reported to affect whitefly infestation on cassava (Uzokwe et al., 2016; MacFadyen et al., 2018; Mugerwa et al., 2019; Kriticos et al., 2020; Kalyebi et al., 2021).

At the regional level, whitefly counts in the South-West, North-West and Littoral Regions were higher than those of the other regions. The high whitefly population in these regions could be partly due to the common cultivation of improved varieties, which have been shown to attract more whiteflies compared to local landraces (Omongo et al., 2012; Kalyebi et al., 2018; Doungous et al., 2021). Moreover, for the Littoral and South-West Regions, the high whitefly abundance could also be related to their high relative humidity and low altitude as these regions are close to the Atlantic Ocean. Whitefly counts decreased with increasing altitude, as also reported in Madagascar and Tanzania by Harimalala et al. (2015) and Szyniszewska et al. (2017), respectively. In the North and Far-North regions, the relative humidity is lower, and the annual mean temperature is higher compared to the other regions, which may be one of the reasons whitefly abundance was lower. Katono et al. (2021) showed that high temperature and low relative humidity had a negative effect on *B. tabaci* abundance on cassava.

Although cutting-derived infection was higher compared to whitefly-derived infection, there was a positive correlation between whitefly presence and disease incidence. Therefore, the absence of whiteflies in the North (Leunda, Poli and Toubaka) and Adamawa (Djalingole, Mayo Baleo and Woulde) Regions and the very low whitefly counts recorded in the remaining healthy farms may contribute to the absence of CMD symptoms. However, the lowest whitefly mean count was recorded on plants with the highest severity score. This could be because virus-infected cassava leaves may be repellent to or present an unattractive environment for whitefly settling as it has been reported in other systems (Wamonje et al., 2020).

Our data highlighted counts of more than 50 adult whiteflies per plant in some surveyed fields. This is alarming since epidemics of CMD and other cassava diseases in Uganda, parts of western Kenya and north-western Tanzania have been associated with similar whitefly populations on cassava (Colvin et al., 2004; Legg et al., 2014b; Mugerwa et al., 2021).

This first countrywide study demonstrates that CMD is widely distributed in Cameroon and that the main source of dissemination is through infected cuttings. To better manage CMD and improve cassava productivity, there is a need for an integrated strategy based on multiplication, distribution, and adoption of improved resistant or tolerant cassava planting materials for local farmers, training of farmers on recognition of the disease and use of healthy cuttings when establishing new plots. The high whitefly count and the exchanges of contaminated planting materials by farmers between regions and countries provide suitable conditions for introduction and expansion of virulent CMG species or strains leading to a pandemic of severe CMD. Future efforts should aim to characterize the viruses and vectors, reinforce phytosanitary and quarantine measures, and implement a frequent surveillance or monitoring program to prevent the spread of CMD and to minimize its impact in order to mitigate possible outbreaks.

## Data availability statement

The data supporting the findings of this study are available from the corresponding author upon request.

## Declaration of competing interest

The authors declare that they have no known competing financial interests or personal relationships that could have appeared to influence the work reported in this paper.
